# Genome-Wide Characterization and Expression Analyses of Major Latex Protein Gene Family in *Populus simonii × P. nigra*

**DOI:** 10.3390/ijms25052748

**Published:** 2024-02-27

**Authors:** Xin Sun, Yao Li, Yao Sun, Qiong Wu, Lei Wang

**Affiliations:** Department of Biotechnology, Institute of Advanced Technology, Heilongjiang Academy of Sciences, Harbin 150001, China; sunxin@cau.edu.cn (X.S.)

**Keywords:** *Populus simonii × P. nigra*, major latex protein, abiotic stress, genome-wide identification, expression profiles

## Abstract

Major latex proteins, or MLPs, are crucial to plants’ capacity to grow, develop, and endure biotic and abiotic stresses. The *MLP* gene family has been found in numerous plants, but little is known about its role in *Populus simonii × P. nigra*. This study discovered and assessed 43 *PtMLP* genes that were unevenly dispersed throughout 12 chromosomes in terms of their physicochemical characteristics, gene structure, conserved motifs, and protein localization. Based on their phylogeny and protein structural characteristics, three separate subclasses of *PtMLP* family were identified. Segmental and tandem duplication were found to be essential variables in the expansion of the *PtMLP* genes. The involvement of the *PtMLP* genes in growth and development, as well as in the responses to different hormones and stresses, was demonstrated by *cis*-regulatory element prediction. The *PtMLP* genes showed varying expression patterns in various tissues and under different conditions (cold, salt, and drought stress), as demonstrated in RNA-Seq databases, suggesting that *PsnMLP* may have different functions. Following the further investigation of the genes demonstrating notable variations in expression before and after the application of three stresses, *PsnMLP5* was identified as a candidate gene. Subsequent studies revealed that *PsnMLP5* could be induced by ABA treatment. This study paves the way for further investigations into the *MLP* genes’ functional mechanisms in response to abiotic stressors, as well as the ways in which they can be utilized in poplar breeding for improved stress tolerance.

## 1. Introduction

Initially identified in 1985 in opium poppies, major latex proteins (MLPs) are a class of tiny proteins specific to plants, with a molecular weight of roughly 20 kDa. As the opium poppy is subjected to mechanical damage, the proteins are secreted to the surface of the wounded plant and are dispersed throughout the growing and mature latex ducts of the plant [1,2]. After their initial identification, MLPs were found in cotton, apple, peach, tobacco, grape, and other plants [2,3,4,5,6,7,8]. Three groups of MLP homologs can be distinguished: pathogenesis-related protein 10 (PR-10), MLPs, and Bet v 1. As PR-10 family members exhibit allergenicity and are present as allergens in many plants, there are more studies on Bet v 1 and PR-10 and fewer on MLPs [9,10]. This is because oral allergy syndrome and other conditions can be caused by cross-reactivity with homologous Bet v 1-specific IgE, which explains why studies on Bet v 1 and PR-10 were conducted earlier and primarily from a medical perspective [11]. As a result, MLPs have received less attention in research in comparison to Bet v 1 and PR-10 [10,12]. However, as MLPs have broader biological activity than the other two homologous proteins, they have attracted increasing attention in recent years.

Members of the homologous family of MLPs have a common Bet v 1 structural domain. Studies have shown that, whereas there is little similarity in the primary structure (amino acid sequence), there is an elevated degree of similarity in the secondary and tertiary structures, which consist of three to four α-helices (short, α1, α2, and long α3), seven β-folds (β1–β7), and nine loops (L1–L9) [13,14]. The most characteristic structural feature of the homolog is an internal hydrophobic cavity with a ‘Y’ shape, which is formed by the β1–β7 wrapped around the long α3 [14,15]. This cavity allows the MLPs in many plants to bind hydrophobic substances, such as steroids, organic compounds, and long-chain fatty acids. MLPs from the Cucurbitaceae family can bind hydrophobic organic pollutants in the roots and then transport them to the aerial parts [15,16]. Cucurbitaceae *MLP* genes have the ability to move hydrophobic organic contaminants that are absorbed by the roots via hydrophobic crevices of the ‘Y’ type, which is important for bioremediation using cucurbits [17].

The second major structural characteristic of MLPs is the presence of a largely homogenous three-dimensional structure, a glycine-rich L4 loop (GXGGXG) connecting β2 and β3. This loop is substantially conserved among MLPs members [14,18]. The steroidogenic acute regulatory protein-associated lipid transfer structural domain, which is the third major structural feature, indicates that MLPs can bind steroids [13]. This steroidal acute regulatory protein-associated lipid transfer domain has also been found in abscisic acid (ABA) receptor pyrabactin resistance 1 (PYR1). As a result, MLPs are probably involved in the ABA signaling pathway through their binding to ABA [19,20].

Members of the *MLP* family are capable of responding to both biotic and abiotic stressors, and they play significant roles in the growth and development of plants [21,22]. The expression of *MLP* genes has been demonstrated to be induced by invasive plant diseases [23,24], and *MLP* genes primarily support plant defense via signals of acquired resistance (SAR) and intrinsic immunity [25,26,27]. Yang et al. discovered that the expression of *GhMLP28* could be induced by exogenous *Verticillium dahliae*, while *GhMLP28* contributed to *Verticillium dahliae*’s defense by promoting GhERF6’s binding to the recognition site in the upstream region of the *PR* gene. *GhERF6* increased the expression of the *PR* gene and conferred disease resistance on cotton by binding to the GCC-box [5]. In the *Arabidopsis thaliana* functionally deficient mutant *mlp*, leaves farther from the inoculation site lost resistance, suggesting that *MLP* genes are involved in plant SAR. In addition, MLPs can increase plant disease resistance by inducing the formation of flavonoid compounds [3].

Drought, low temperatures, salt, and alkalinity are types of abiotic stress that can inhibit photosynthesis, create metabolic problems, impede growth and development, and, in extreme cases, even cause mortality. Effector molecules, which are directly involved in metabolism, and their upstream regulators enable plants to respond to abiotic stressors. Few research studies have been performed to investigate the ways in which MLPs affect abiotic stress in plants; however, in recent years, an increasing number of studies have suggested that MLPs play an essential role in this context. A cold stress response element, an SA-induced element, a salt stress response element, and an injury response element are all found in the promoter region of *GhMLP* [5]. The promoters of grape *VvMLP* and tobacco *MLP28* contain abiotic stress response elements, such as low-temperature-, light-, dehydration-, and hormone-related response elements, including gibberellin (GA), ethylene (ET), growth hormone, and ABA [6,22]. According to Wang et al. [28], *Arabidopsis thaliana MLP43* functions as a positive regulator and can bind to SnRK2 and the ABA response element-binding factor ABF1. It additionally plays a role in the ABA signaling-mediated regulation of plant drought tolerance. Furthermore, it has been shown that the expression of certain homologous *MLP* genes can be induced by salt treatment, which improves plant salt tolerance. According to Yuan et al. [29], ABA, NaCl, and drought stress are capable of inducing some of the *MLP* genes in apples. These genes may also be implicated in the response of apples to abiotic stresses by way of PRSP, SNRK1/2, bHLH, and other mechanisms. Liu et al. [7,30] also demonstrated that tobacco’s cold tolerance was enhanced by *NtMLP423* overexpression. The results of the above research imply that MLPs play a role in how plants react to abiotic stressors.

A hybrid of *Populus nigra* L. and *Populus simonii* Carr, *Populus simonii × P. nigra* is a fast-growing, adaptable, cold- and drought-resistant species that is important for greening, afforestation, and timber in China. It is also a good material for the study of genetic engineering and forest physiology. The whole-genome sequencing and annotation of *Populus simonii × P. nigra* has not yet been completed; however, the genome of the model species, *Populus trichocarpa*, has been obtained, and its whole-genome sequencing and annotation were performed in 2006 [31], providing a basis for the genome-level analysis of *Populus simonii × P. nigra*. In this study, the genome of *Populus trichocarpa* was utilized for genome-wide identification and analysis. The main elements affecting the yield and quality of poplar are abiotic stresses, including low temperature, high temperature, salt and drought stress, etc. Characterizing and researching the *MLP* gene family in the poplar genome is very important since it has been demonstrated that this family is involved in the plant’s response to abiotic stresses but it has not yet been identified and functionally studied in poplar.

In this context, we identified 43 *MLP* genes in *Populus trichocarpa* and thoroughly investigated their physicochemical characteristics and conserved motifs, the *cis*-regulatory elements in the promoter region, chromosomal localization, protein localization, gene duplication events, and evolutionary relationships. We further explored the expression patterns of the *PsnMLP* genes in different tissues and under different abiotic stresses (drought, salt stress, and cold stress). Furthermore, we cloned *PsnMLP5*, the gene that changed in the *Populus simonii × P. nigra* roots most significantly and concurrently in response to all three stresses, and we examined its structure and possible relationship with ABA. Although the MLPs have been identified in some species of poplar [32], this work is the first in which the *MLP* gene family has been identified and characterized in *Populus simonii × P. nigra*, laying the foundation for the further investigation of the functional mechanisms of the *MLP* genes in response to abiotic stresses in *Populus simonii × P. nigra*, as well as their application in the breeding of poplar for stress tolerance.

## 2. Results

### 2.1. Identification and Characterization of PtMLP Genes

**Figure 1:**
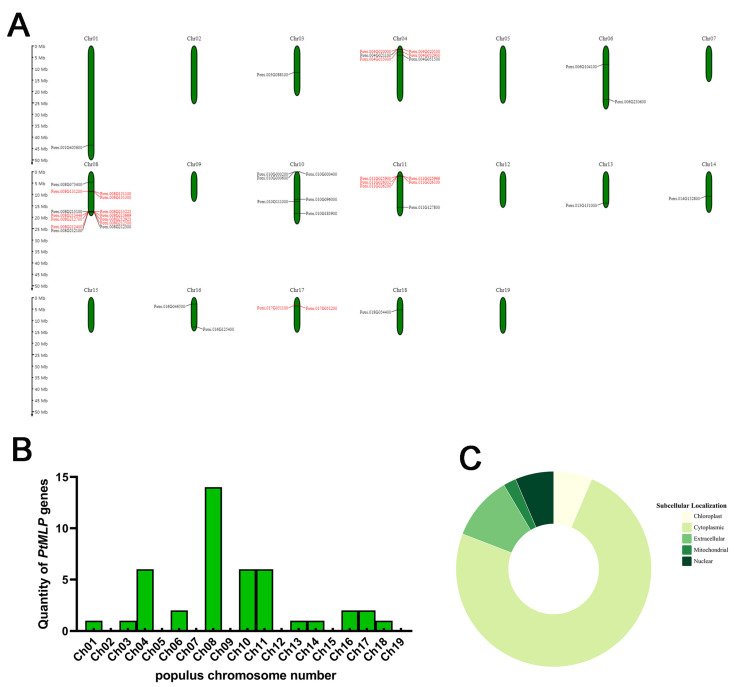
Genomic distributions and prediction of subcellular localization of PtMLPs. (**A**) *PtMLP* gene distribution across 19 chromosomes of *Populus trichocarpa*. Vertical bars represent chromosomes and the scale on the left represents the chromosome length (Mb). Tandemly duplicated genes are indicated in red. (**B**) Numbers of *PtMLP* genes on each chromosome. (**C**) Subcellular localization prediction of PtMLPs.

A total of 20 *PtMLP* genes were initially screened using the Bet_v_1 structure file, and 46 *PtMLP* genes were identified after sequence alignment and re-modeling. Using SMART and Interpro, we verified that all of the identified genes encoded proteins with a Bet_v_1 domain. A total of 43 *MLP* genes in *Populus trichocarpa* were obtained at the genome-wide level and named *PtMLP1–PtMLP43* for brevity. The members of the *PtMLP* gene family varied widely in terms of their amino acid lengths and physicochemical properties. The number of amino acids ranged from 79 (*PtMLP21*) to 221 (*PtMLP41*), the molecular weight of the proteins ranged from 8471.61 (*PtMLP21*) to 24,059.16 Da (*PtMLP9*), the theoretical isoelectric point ranged from 4.65 (*PtMLP7*) to 8.73 (*PtMLP9*), the instability coefficients ranged from 16.92 (*PtMLP14*) to 54.69 (*PtMLP31*), the aliphatic index ranged from 74.9 (*PtMLP38*) to 109.21 (*PtMLP37*), and the overall average value of hydropathicity ranged from −0.522 (*PtMLP38*) to 0.028 (*PtMLP37*). The predictive analysis of subcellular localization showed that the PtMLPs were mostly localized in the cytoplasm (79.1%), and very few were localized in the extracellular region (11.6%), nuclear region (6.98%), and organelles, such as chloroplasts (6.98%) and mitochondria (2.33%) (Figure 1C). The details are listed in Table S1.

### 2.2. Chromosome Distribution and Phylogenetic Analysis of the PtMLP Gene Family

The 43 *PtMLP* genes were distributed extensively and unevenly on 12 chromosomes, but there were no *PtMLP* genes located on chromosomes 2, 5, 7, 9, 12, 15, and 19. A total of 14 *PtMLP* genes were located on chromosome 8, representing the largest number of *PtMLP* genes. Most *PtMLP* genes were located at the proximal or distal ends of these chromosomes, and high densities of *PtMLP* genes were distributed at the front ends of chromosomes 4 and 11 and the bottom of chromosome 8 (Figure 1A,B).

**Figure 2:**
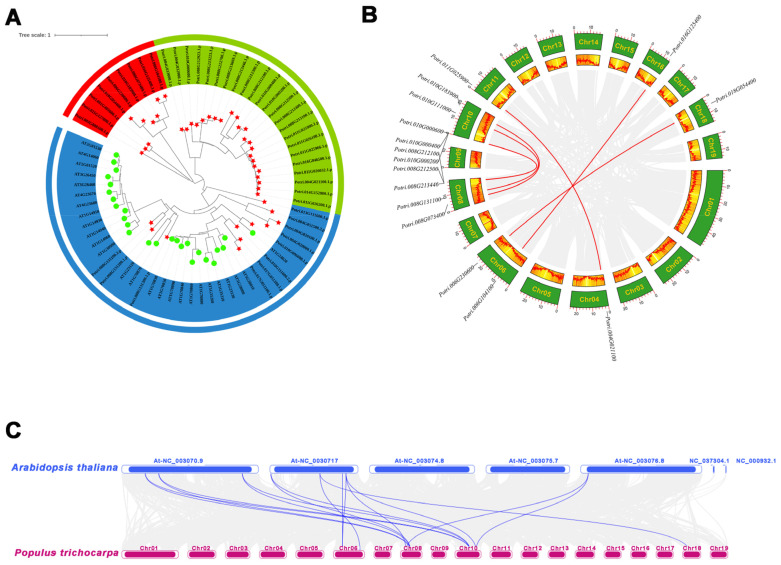
Phylogenetic analysis of PtMLPs. (**A**) Phylogenetic relationships of MLPs between *Populus trichocarpa* and *Arabidopsis thaliana*. The three subclasses are marked with different colors. Red represents Class I, blue represents Class II, and green represents Class III. PtMLPs are marked with red stars and AtMLPs are marked with green circles. (**B**) The segmental replication events of *PtMLP* genes in *Populus trichocarpa*. Gray lines indicate all synteny blocks in the *Populus trichocarpa* genome and red lines indicate segmental duplication of *PtMLP* genes. (**C**) Collinearity analysis of *MLP* genes between *Populus trichocarpa* and *Arabidopsis thaliana*. Blue lines indicate the homologous *MLP* gene pairs.

To investigate the evolutionary relationships among the *MLP* genes from *Arabidopsis thaliana* and *Populus trichocarpa*, an unrooted phylogenetic tree was generated based on the alignment of the amino acid sequences for 69 MLPs, including 26 Arabidopsis and 43 poplar members. The results showed that the *MLP* gene family could be divided into three subfamilies, namely, Class I, Class II, and Class III, containing 9, 11, and 23 *PtMLP* family members, respectively. The evolutionary tree showed that *PtMLP* members belonging to subfamily Class II were closer to the Arabidopsis *MLP* family than the other two subfamilies (Figure 2A).

### 2.3. Gene Structure and Conserved Motifs of the PtMLP Gene Family

**Figure 3:**
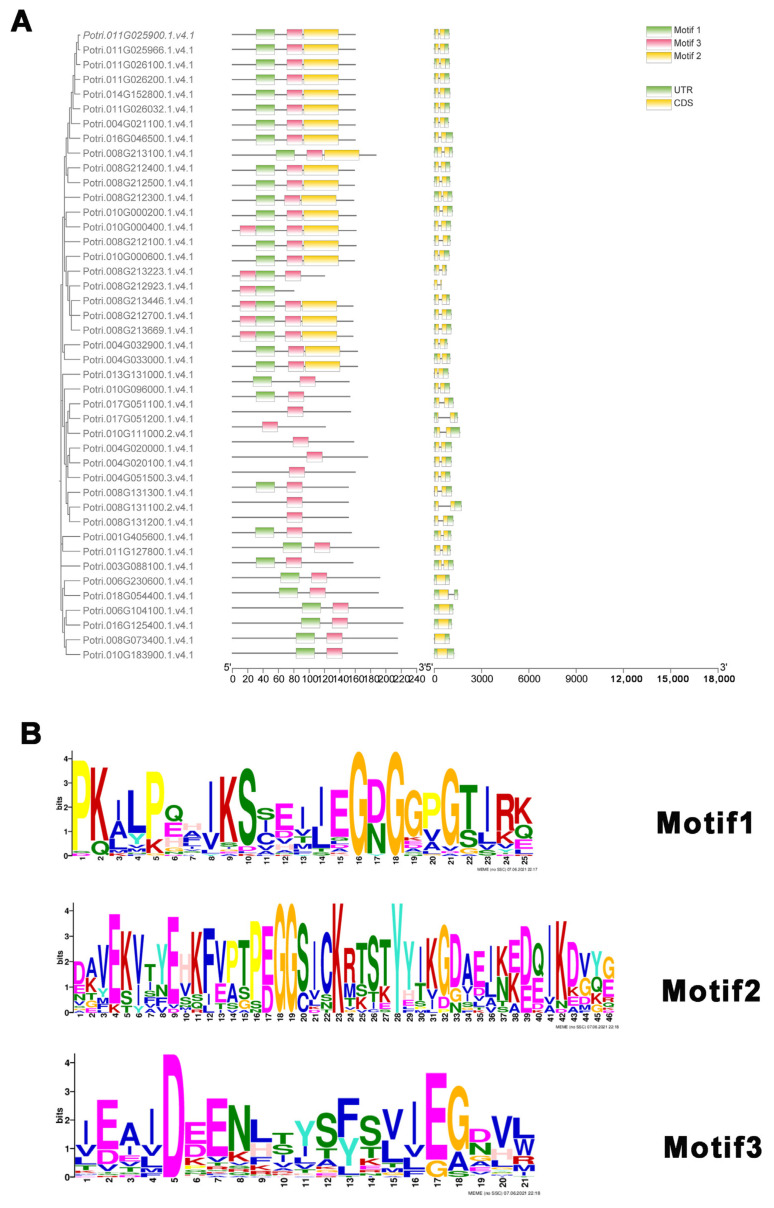
Gene structure and conserved motifs of PtMLP family. (**A**) The gene structure and conserved motifs of PtMLPs. Left: Conserved motifs are displayed in different colors and correspond one-to-one in the structural diagram. Right: yellow boxes represent exons, green boxes represent untranslated regions, and black lines indicate introns. (**B**) The logo and indicated domain of each motif.

The exon and intron composition analysis revealed that most members of the *PtMLP* gene family had two exons and only a small percentage had one exon, all belonging to Class I (Figure 3A). Following the motif analysis, three conserved motifs were found in the *PtMLP* family. Members of Class I had two conserved motifs (motif1 and motif3). Most members of Class II had only one conserved motif (motif3), with the exception of *PtMLP3*, which had two conserved motifs but belonged to Class II. Members of Class III had three conserved motifs (Figure 3A,B). These results indicate that genes with closer kinship have higher conserved motif similarity; however, there are some distinctions between individual genes. Additionally, family members with comparable gene structures can be aggregated into a single group.

### 2.4. Gene Duplication, Genome Synteny and Selective Pressure Analysis of the PtMLP Gene Family

Tandem duplications and segmental duplications have promoted the expansion of new gene family members and the generation of new functions in the evolution of plant genomes. The segmental and tandem duplication events in the *PtMLP* gene family were investigated to elucidate its gene duplication events in *P. trichocarpa*. The analysis showed that there were 13 pairs of tandemly duplicated genes (21/43, 48.8%) in the *PtMLP* family: one pair on chromosome 17, two pairs on chromosome 4, three pairs on chromosome 11, and seven pairs on chromosome 8 (Figure 1 and Table S2). While the CDS sequences differed, the amino acid sequences of *PtMLP32* and *PtMLP33* were the same. *PtMLP20*, *PtMLP24*, and *PtMLP25* were identical in both amino acid sequences and CDS sequences. Using MCScanX techniques, eight segmental duplication occurrences (16/44, 36.4%) were found, in addition to tandem duplication (Figure 2B and Table S2). The results demonstrate that replication events, in which tandem and segmental duplications are major contributors, are probably responsible for the production of *PtMLP* family members.

**Figure 4:**
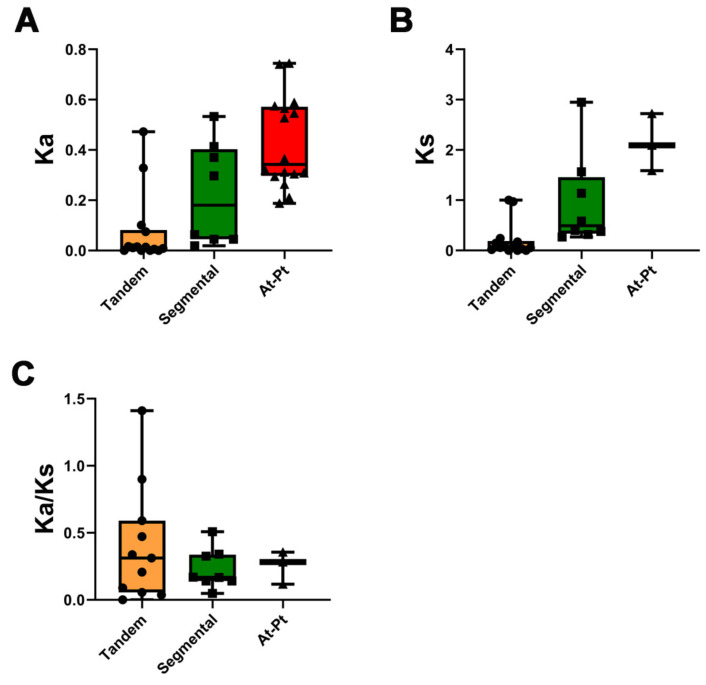
Ka/Ks analysis of the *PtMLP* gene family. (**A**) Average Ka values of tandem duplication and segmental duplication of *PtMLP*s and the duplication between *Populus trichocarpa* and *Arabidopsis thaliana*. (**B**) Average Ks values of tandem duplication and segmental duplication of *PtMLP*s and the duplication between *Populus trichocarpa* and *Arabidopsis thaliana*. (**C**) Average Ka/Ks values of tandem duplication and segmental duplication of *PtMLP*s and the duplication between *Populus trichocarpa* and *Arabidopsis thaliana*. The black dots, triangles and squares represent the data values of tandem, segmental and At-Pt duplication, respectively.

The rate of non-synonymous (Ka) and synonymous (Ks) substitutions is important in understanding the repeated event selection pressure. A Ka/Ks value of >1 indicates that positive selection is performed, a Ka/Ks value of 1 indicates neutral evolution, and a Ka/Ks value of <1 indicates purifying selection. The Ka/Ks values of tandemly duplicated *PtMLP* genes were 0–1.410, with a mean value of 0.401, and one pair had a value of >1, which was likely to have received positive selection (*PtMLP42*, *PtMLP43*). The Ka/Ks values of segmentally duplicated *PtMLP* genes ranged from 0.04 to 0.51, with an average value of 0.230. From the results, it is clear that most of the *PtMLP* family received purifying selection (Figure 4 and Table S3). To further explore the possible evolutionary processes of the *PtMLP* genes, we analyzed the collinearity of the *MLP* family genes in *Populus trichocarpa* and *Arabidopsis thaliana*. The results showed that a total of 16 pairs of orthologs were identified, with a Ka/Ks value of 0.117–0.356 (Figure 2C and Figure 4, and Table S3).

### 2.5. Cis-Regulatory Element Analysis of PtMLP Gene Family

**Figure 5:**
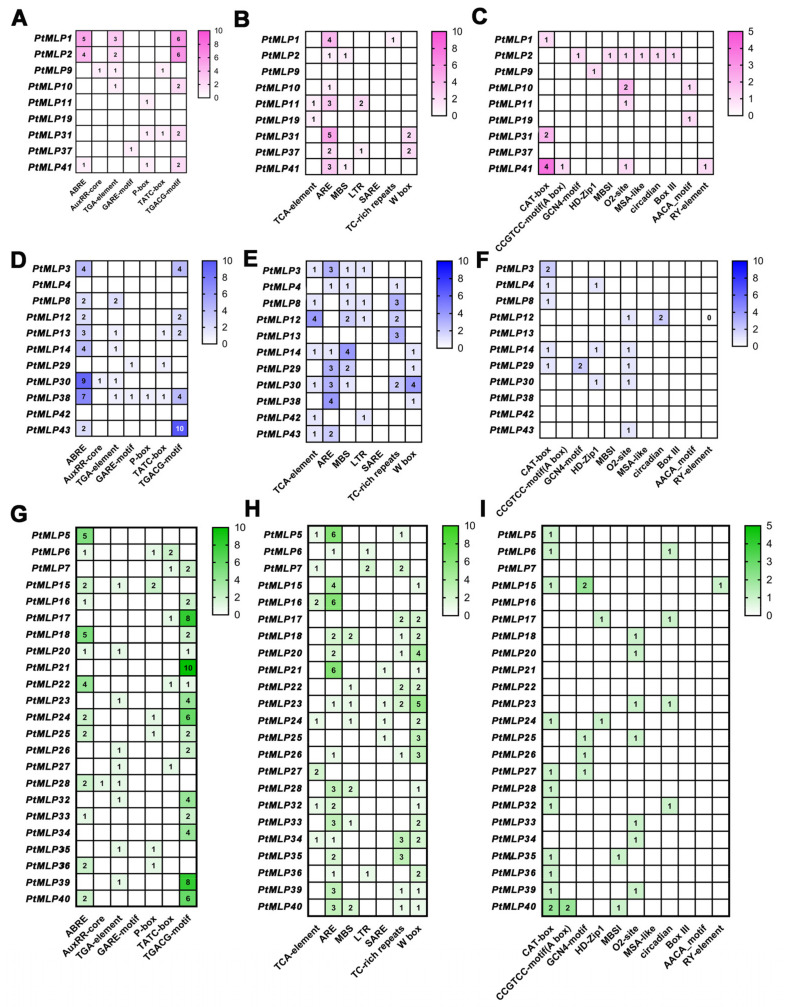
Promoter *cis*-regulatory element analysis of the *PtMLP* gene family. (**A**–**C**) *Cis*-regulatory elements of *PtMLP*s in Class I. (**A**) represents the hormone-responsive elements, (**B**) represents the abiotic- and biotic-stress-responsive elements and (**C**) represents the growth-and-development-related elements. (**D**–**F**) *Cis*-regulatory elements of *PtMLP*s in Class II. (**D**) represents the hormone-responsive elements, (**E**) represents the abiotic- and biotic-stress-responsive elements and (**F**) represents the growth-and-development-related elements. (**G**–**I**) *Cis*-regulatory elements of *PtMLP*s in Class III. (**G**) represents the hormone-responsive elements, (**H**) represents the abiotic- and biotic-stress-responsive element and (**I**) represents the growth-and-development-related elements.

The *cis*-regulatory elements within the first 2000 bp fragments upstream of the *PtMLP* genes were predicted and summarized. These elements were categorized into three main groups based on their functions: hormone-responsive, abiotic- and biotic-stress-responsive, and growth-and-development-related elements. *PtMLP30* had the most *cis*-regulatory elements (24), while *PtMLP19* and *PtMLP42* had the fewest (2). Hormone-responsive elements were the most common *cis*-regulatory elements in Class I and Class II (44.2% and 46.3%), while abiotic and biotic stress-responsive elements (45.9%) were the most common elements in Class III, and growth-and-development-related elements were the least common in all three subfamilies. The majority of Class I members possessed both the ARE element and the TGACG-motif element (methyl jasmonate response elements). The TGACG-motif element was the most common hormone-responsive element (18), and the ARE element was the most common stress-responsive element (19). The most common hormone-responsive element among the Class II members was the ABRE element (33), while the most common stress-responsive element was the ARE element (17). The majority of members had the ABRE element. With regard to Class III members, the most common hormone-responsive element was the TGACG-motif (64), the most common stress-responsive element was the ARE element (47), and the majority of members had both the TGACG-motif and W-box elements. The most common growth-and-development-related element in all three subfamilies was the CAT-Box, and only Class I members had the AACA-motif element (Figure 5).

### 2.6. Expression Patterns of PsnMLPs in Different Tissues

**Figure 6:**
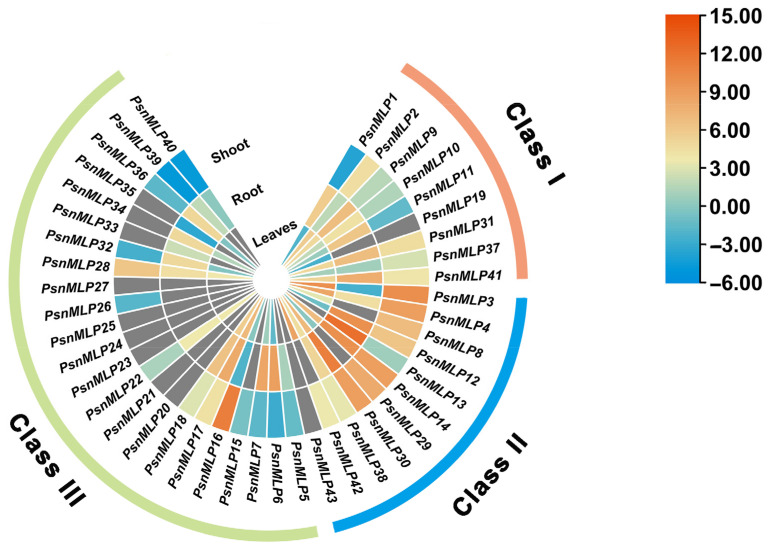
Expression patterns of *PsnMLP* genes in different tissues. The gray color indicates that the expression of the gene could not be detected in the RNA-Seq.

We performed RNA-Seq analysis using *Populus simonii × P. nigra* as the plant material and the *Populus trichocarpa* genome as the reference genome. Based on the 43 *PtMLP* genes identified from the results above, the *MLP* genes of *Populus simonii × P. nigra* were named *PsnMLP1–PsnMLP43*. In comparison to the leaves and stems, the majority of the *PsnMLP* genes were significantly expressed in the roots. *PsnMLP13* had the greatest expression level, while seven genes (*PsnMLP20*, *PsnMLP21*, *PsnMLP23*, *PsnMLP24*, *PsnMLP25*, *PsnMLP27*, *PsnMLP43*) were expressed at extremely low levels in all tissues (Figure 6). Members of Class I were expressed in the roots, stems, and leaves, with higher expression in the roots than in the stems and leaves. Most of the Class II members were expressed in all three tissues and at a higher level. Many of the Class III members had very low expression, being nearly undetectable, and the majority of them were expressed in the roots. It is notable that *PsnMLP19* was specifically expressed in the leaves, while *PsnMLP15* and *PsnMLP26* were expressed only in the stems and *PsnMLP35* was expressed in the roots only, suggesting that these genes are involved in the regulation of leaf, stem, and root growth and development, respectively.

### 2.7. Expression Patterns of PsnMLP Genes Response to Different Abiotic Stresses

As the *PsnMLP* genes were mainly expressed in the roots, and *cis*-regulatory elements, such as those responsive to low temperatures and drought, were prevalent in the promoters of the *PtMLP* genes, the expression patterns under drought, cold, and salt stress in the roots were analyzed using RNA-Seq to further explore the functions of the *PsnMLP* genes.

**Figure 7:**
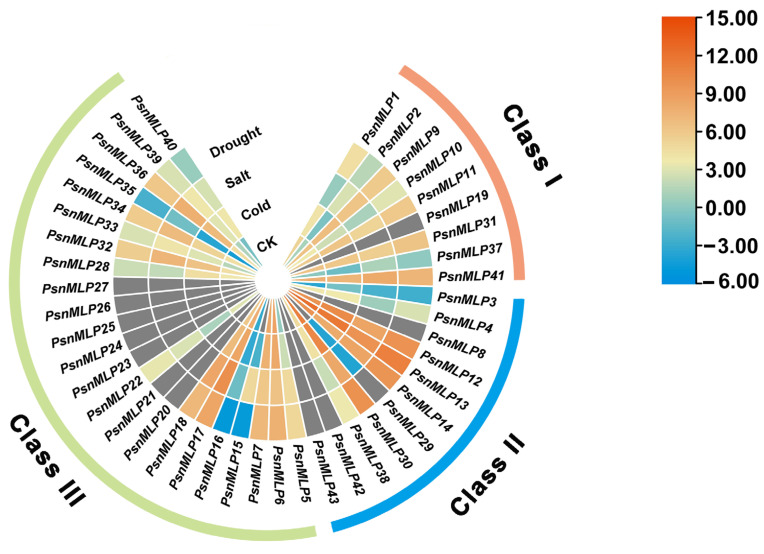
Expression patterns of *PsnMLP* genes in response to different abiotic stresses. The gray color indicates that the expression of the gene could not be detected in the RNA-Seq.

With the exception of *PsnMLP9* and *PsnMLP11*, which displayed slight upregulation under cold stress, all of the genes in Class I had downregulated expression. With the exception of *PsnMLP2* and *PsnMLP37*, all genes were downregulated in response to salt stress. In response to drought stress, the expression of five genes was downregulated and that of four genes was upregulated. Under each of the three stresses, the expression levels of *PsnMLP1*, *PsnMLP10*, and *PsnMLP31* were downregulated. Members of the Class I family showed lower expression overall following their exposure to the three stressors (Figure 7).

With the exception of *PsnMLP3*, *PsnMLP12*, and *PsnMLP29*, which were upregulated, all Class II genes underwent downregulation in response to cold stress. *PsnMLP3* and *PsnMLP29* were upregulated and the others were downregulated under salt stress; similarly, *PsnMLP3* was upregulated and the others were downregulated under drought stress. In all three situations, the comparatively low expression of *PsnMLP3* was upregulated (Figure 7).

With the exception of *PsnMLP6*, *PsnMLP7*, and *PsnMLP16*, whose expression was downregulated, the expression of members of the Class III family was generally upregulated following abiotic stress. It is evident that several subfamily members react to abiotic stress in different ways, indicating that the various subfamily members may perform different functions in response to abiotic stress (Figure 7).

**Figure 8:**
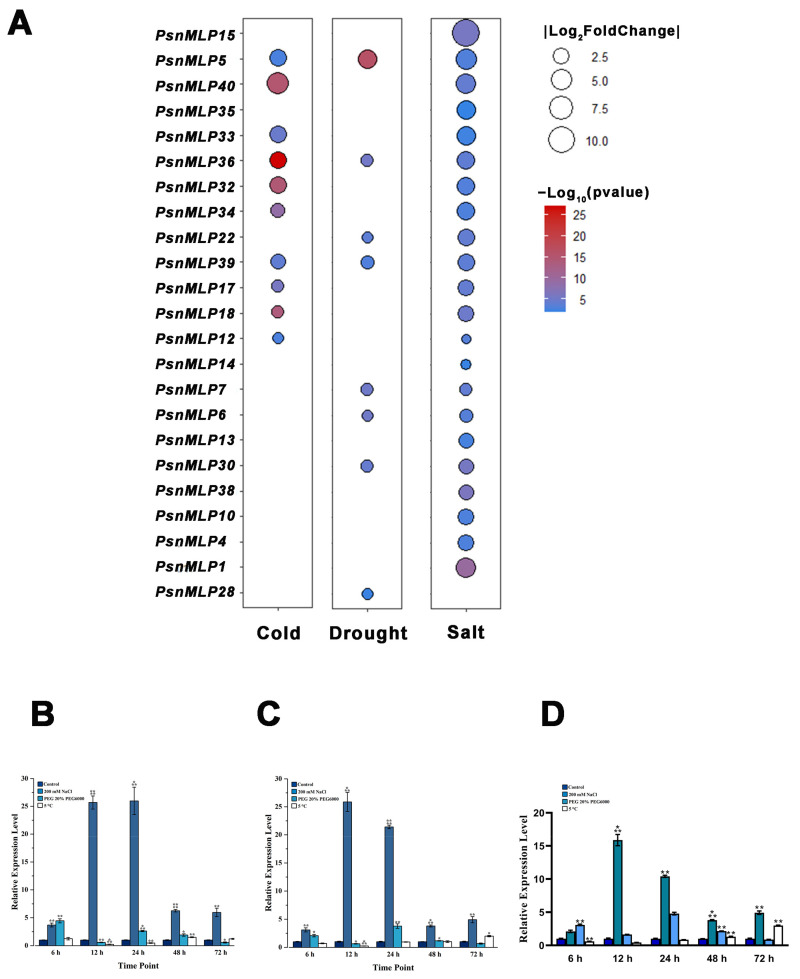
Expression analysis of *PsnMLP* genes with significant differences in expression levels. (**A**) *PsnMLP* genes with significant variations in expression under abiotic stress. The size of the circle represents the absolute value of log_2_fold change. The color of the circle indicates the q-value. (**B**–**D**) qRT-PCR results of *PsnMLP5* (**B**), *PsnMLP36* (**C**) and *PsnMLP39* (**D**). * represents *p* < 0.05, ** represents *p* < 0.01, *** represents *p* < 0.001, **** represents *p* < 0.0001.

A total of 23 *PsnMLP* genes exhibited significant variations in expression under abiotic stress. The gene with the largest change in expression was *PsnMLP15* (log_2_foldchange = 10.59), and the gene with the smallest change in expression was *PsnMLP12* (log_2_foldchange was −1.010) (Table 1). Following treatment with salt stress, a total of 22 *PsnMLP* genes (of which 12 were activated and 10 were repressed) exhibited substantial differences regarding changes in their expression. Among them, all 12 *PsnMLP* genes activated by salt stress were from Class III. In contrast, two of the salt-stress-suppressed *PsnMLP* genes were from Class I, with two from Class II, and six from Class III. Following drought stress, the expression of eight *PsnMLP* genes significantly varied; four of these genes were activated by drought stress and the remaining four were repressed. With the exception of *PsnMLP30* from Class II, all of them were members of Class III. Following cold stress treatment, the expression of ten distinct *PsnMLP* genes changed significantly. All of the differential expression genes were induced by cold stress and belonged to Class III, except *PsnMLP12* (Figure 8).

Three genes, *PsnMLP5*, *PsnMLP36*, and *PsnMLP39*, responded to the three abiotic stresses simultaneously. These genes were all from Class III and were all induced by the three different abiotic stresses. *PsnMLP5* displayed the greatest change in expression; thus, we selected it as a candidate gene for further investigation. We next chose all three *PsnMLP* genes for the qRT-PCR validation of the RNA-Seq data, which demonstrated the plausibility of the RNA-Seq results (Figure 8).

### 2.8. Response to ABA of PsnMLP5

**Figure 9:**
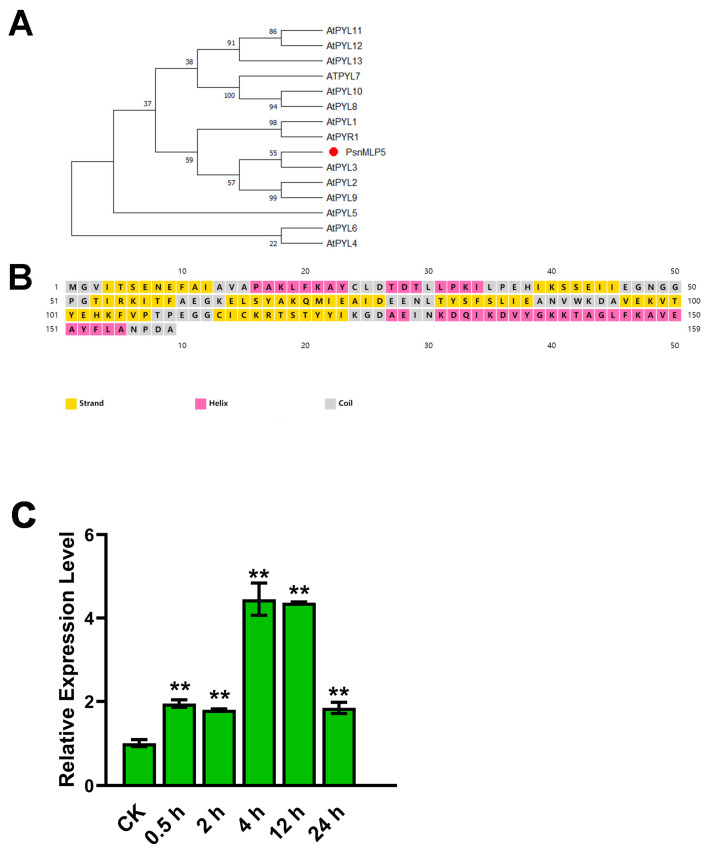
Response to ABA of *PsnMLP5.* (**A**) Evolutionary analysis between PsnMLP5 and AtPYR/PYL receptors. (**B**) Secondary structure of PsnMLP5. (**C**) Expression level of *PsnMLP5* under ABA treatment. ** represents *p* < 0.01.

We cloned *PsnMLP5* and proceeded with the analysis. According to a homology study, *AT1G24020* (*MLP423*) is the Arabidopsis homolog of *PsnMLP5*, and according to GO annotation, AtMLP423 possesses ABA-binding, protein-phosphatase-inhibitory, and ABA receptor activity. The PYR/PYL family of ABA receptors and the MLP family are both protein families with START domains and belong to the START protein superfamily. In addition, upon examining PsnMLP5’s secondary structure, we discovered that it had four α-helices and seven β-folds, a structure shared by the PYR/PYL proteins. The sequence similarity between PsnMLP5 and the AtPYR/PYL receptors was also demonstrated by evolutionary studies, indicating that PsnMLP5 is likely to function as a type of ABA receptor, as well as playing a role in the ABA signaling pathway to regulate the poplar response to salt stress by binding ABA (Figure 9). Consequently, we postulated that *PsnMLP5* would respond to ABA. Therefore, we sprayed poplar seedlings with an ABA solution and then detected the expression of *PsnMLP5*. The results showed that *PsnMLP5* could be induced by ABA, and its expression was upregulated maximally in the 4th hour of treatment and then gradually decreased, but it always remained upregulated, which proved that the gene was responsive to ABA.

## 3. Discussion

MLPs are a family of plant-specific proteins that have been demonstrated to play significant roles in both abiotic and biotic stress responses, as well as plant growth and development [9,33,34]. Although MLPs have been found in a wide variety of plants, and some of these have been subjected to related gene family investigations, for poplar, a model plant used in forest tree genomics research, there are not yet any published *MLP* genes. In this study, the *PtMLP* genes were screened and identified at the genome-wide level. A total of 43 *PtMLP* genes were found, and, using bioinformatics techniques, their physicochemical characteristics, gene structures, conserved motifs, protein localization, evolutionary relationships, and promoter *cis*-acting elements were investigated. In addition, extensive research was carried out on the changes in *PsnMLP* gene expression in various tissues, as well as in response to low temperature, drought, and salt stress. Finally, *PsnMLP5*, a gene that responds to these three abiotic stresses at the same time, was screened to investigate its role in abiotic stress and its relationship with ABA. This work provides insights into the role of poplar *MLP* genes in response to abiotic stimuli and provides the information needed to fully understand the poplar *MLP* gene family.

In several plants, the *MLP* gene family has been fully described at the genome level; the number of *MLP* genes differs among species but not based on the genome size. In the dicotyledonous plant tomato (827 M), 34 were found, as well as 36 in apple (700 M) and 14 in grape (490 M) [3,6,28,29]. However, only two to three *MLP* genes were identified in the monocotyledons *Brachypodium distachyon* and *Zea mays* [9]. The difference in the number of *MLP* genes in monocots and dicots may indicate that the *MLP* family diverged after the monocotyledon–dicotyledon division during plant evolution. In this research, we identified a total of 43 *PtMLP* genes in the *Populus trichocarpa* genome, which is consistent with the results of previous studies. The 43 *PtMLP* genes can be classified into three subfamilies, and members of the same subfamily have similar gene structures and conserved motifs. Physicochemical and subcellular localization analyses showed that most of the *PtMLP* family was located in the cytoplasm, with a small portion in the nucleus and extracellular region, which is also consistent with previous studies [35].

Gene duplication is important for the evolution of physiological and ecological diversity in plants [36]. In this study, the *PtMLP* gene family experienced 13 tandem duplication pairs and eight segmental duplication pairs, with the majority of the duplication events occurring at the ends of the chromosomes. This result was consistent with earlier research on *MLP* genes in tomato and peanut, which revealed that some *MLP* genes were concentrated at the chromosomal ends [35,37]. These findings indicate that replication events, in which tandem and segmental duplications play a major role, are likely to be necessary for the generation of *MLP* family members.

*Cis*-acting promoter elements are involved in the regulation of gene expression through interactions between promoter-binding sites and transcription factors [2,38,39]. The majority of the *PtMLP* gene promoter regions in our study had a great number of ARE and ABRE elements; this finding is consistent with previous research on *MLP* genes in other plants [5,6]. The promoters of the *MLP* genes in peanuts and apples both had more ARE and ABRE motifs, indicating that the *MLP* genes are probably responsive to ABA and implicated in oxidative stress [29,37]. It is interesting to note that, in contrast to earlier research, the current study identified a large number of TGACG-motifs in the promoter regions of *PtMLP*s. Me-JA, similar to ABA, has been suggested to be involved in plant growth and development, especially in response to abiotic stresses such as cold stress, salt stress, and others [40]. These results suggest that the *PtMLP* genes play an important role in the plant response to abiotic stress.

MLPs are thought to be involved in the regulation of growth and developmental processes in plants, and MLP family members have different expression patterns in the different tissues of plants. Studies on tomato showed that ten members of the *SlMLP* family were highly expressed in the roots, four in the stems, six in the leaves, eight in the flowers, five in the fruits, and six in the seeds [35]. Studies on grape found that most of the *VvMLP*s were highly expressed in the roots as well as in the mature leaves, with lower levels in the stems [6]. In this research, the majority of the *PtMLP* genes were expressed in the roots; nevertheless, there were variations across the various subfamilies. The genes in Class II were expressed in the roots, stems, and leaves at higher levels than in the other two subfamilies. Furthermore, a few members were exclusively expressed in a particular tissue, indicating that distinct subfamilies might play distinct roles in poplar development and growth.

Although the majority of studies on the MLPs’ response to stress focused on biotic stresses, such as disease, a growing number of studies have also shown that MLPs are involved in plants’ responses to abiotic stress in recent years. Heat, cold, and salt stress all induced the expression of grape *VvMLP1/2/3/6/9* [6]. In tomato, *SIMLPs* could respond to low temperature, high temperature, salt, and drought stress, but the number of upregulated *SIMLP*s was low [35]. This research revealed that *PsnMLP* genes belonging to distinct subfamilies had distinct stress response patterns. Specifically, the expression of Class I and II *PsnMLP* genes was primarily repressed in response to abiotic stimuli, while that of Class III *PsnMLP* genes was primarily promoted. Furthermore, the results demonstrated that the majority of the *PsnMLP* genes from Class III that had notable variations in expression were induced following abiotic stress, which suggests that the different subfamilies may exert different functions in response to abiotic stresses. The GO and KEGG analyses indicated that the differential genes were involved in the plant MAPK signaling pathway and phytohormone signaling. Members of Class III may play important roles in the response to abiotic stress in poplar, and their roles may be realized by affecting the MAPK signaling pathway and hormone signaling pathway.

The tertiary structure of the MLPs is similar to that of the ABA receptor, which means that it is likely to have a similar function in binding ABA or participating in the ABA signaling pathway [19,41]. Previous studies have shown that MLPs can participate in ABA synthesis and signaling pathways [28,30]. In our study, *PsnMLP5*, a gene that responds to three abiotic stresses simultaneously, was screened in the roots of *Populus simonii × P. nigra*.

Subsequent investigations revealed that ABA could stimulate *PsnMLP5* expression, indicating that *PsnMLP5* may participate in the ABA signaling pathway [28]. Additionally, its *Arabidopsis* homolog, AtMLP423, was shown to have ABA-binding and receptor activity. Thus, we hypothesize that PsnMLP5 functions as an ABA receptor and participates in the ABA signaling pathway, regulating poplar’s tolerance to abiotic stress. Based on prior research, which also demonstrated that MLPs can use their cavity structure to bind hydrophobic substances for transport from roots to shoots [17], we hypothesize that PsnMLP5 may bind ABA in plant roots and transfer it to other organs via xylem and phloem conduits, regulating the tolerance of the plant to stress. We will take *PsnMLP5* as a candidate gene in subsequent studies to investigate its interactions with ABA and the molecular mechanism involved in regulating abiotic stress resistance in poplar.

## 4. Materials and Methods

### 4.1. Plant Materials and Treatments

The plant materials used in this research were derived from *Populus simonii × P. nigra*, which was obtained from the Northeast Forestry University. Branches from the same asexual line were hydroponically cultivated in a greenhouse and then divided into four groups after new roots, stems, and leaves had grown within two months. These branches were treated with NaCl (200 mM) and PEG-6000 (20.0% *w*/*v*) at 5 °C for 48 h and then frozen in liquid nitrogen immediately and stored at −80 °C for RNA extraction and gene expression analysis.

For the ABA treatment, sterile seedling leaves were sprayed with ABA (Sigma-Aldrich, St. Louis, MO, USA) (100 μM with 0.5% Triton-100 (Thermo Fisher, Waltham, MA, USA)) until dripping for 24 h. Samples (roots, stems, and leaves) were harvested at 0 h, 0.5 h, 2 h, 4 h, 12 h, and 24 h and then frozen in liquid nitrogen and stored at −80 °C for subsequent experiments.

For real-time PCR, the materials and treatments were the same as for the RNA-Seq. The samples were harvested at different time points (0 h, 6 h, 12 h, 24 h, 48 h, and 72 h) and then frozen in liquid nitrogen immediately and stored at −80 °C for expression analysis.

### 4.2. Identification of PtMLP Genes in Populus trichocarpa

The gene files of *Populus trichocarpa* and *Arabidopsis thaliana* were obtained from JGI Phytozome 14.0 (https://phytozome-next.jgi.doe.gov/, accessed on 2 May 2023) [42], and the Hidden Markov Model profiles of Bet_v1 were accessed from the Pfam database (https://pfam.xfam.org/, accessed on 2 May 2023) [43], which was used as a query. Hmmsearch [44] was used to search the Bet_v1 domain against the protein sequences of the *Populus trichocarpa* genome, and the initially screened proteins were subjected to multiple sequence alignment using ClustalOmega (https://www.ebi.ac.uk/Tools/msa/clustalo/, accessed on 3 May 2023). Based on the alignment results, the hmmbuild algorithm was used to construct a more accurate Hidden Markov Model for subsequent querying. Repeating the previous operation using the new model, the MLPs of *Populus trichocarpa* were screened twice to obtain the final protein sequences. After removing all redundant sequences, candidate family members were submitted to Interpro (https://www.interprobps.com/, accessed on 5 May 2023) and SMART (http://smart.embl-heidelberg.de/, accessed on 5 May 2023) to manually screen members with the Bet_v1 domain for further analysis. All identified putative PtMLPs were named after *Populus trichocarpa* with the prefix “Pt”, followed by Arabic numerals sequentially starting from 1.

### 4.3. Characterization of PtMLP Proteins and Genes

The number of amino acids, the molecular weight (MW), and the theoretical isoelectric point (PI) were calculated using the ExPasy site (https://www.interprobps.com/, accessed on 7 May 2023) [45]. The motif analysis was performed using the MEME program (version 5.0.4, http://alternate.meme-suite.org/tools/meme, accessed on 7 May 2023) [46] with the following parameters: the maximum number of motifs was five, the optimum width of motifs was set between 10 and 50, and the distribution of motif occurrences was zero or one per sequence. The subcellular localization analysis of PtMLPs was predicted using WolF PSORT (https://www.genscript.com/wolf-psort/html, accessed on 10 May 2023). The gene structure analysis was performed using TBtools [47].

### 4.4. Chromosomal Localization and Phylogenetic Analysis of PtMLP Gene Family

Chromosome information and gene density information were extracted based on the gene GFF files from genome of *Populus trichocarpa*. MG2C (http://mg2c.iask.in/, accessed on 10 May 2023) was used to generate the chromosomal position map of the *PtMLP* gene family. The full-length protein sequences of the MLPs identified in *Populus trichocarpa* and 26 MLPs in *Arabidopsis thaliana* were used for the phylogenetic analysis. Multiple sequence alignments were performed using the ClustalW process. An unrooted maximum likelihood phylogenetic tree was constructed using MEGA 7.0 software with a bootstrap test with 1000 iterations [48].

### 4.5. Promoter Cis-Regulatory Element and Collinearity Relationship Analysis of the PtMLP Gene Family

Sequences of 2000 bp from the promoters of the *PtMLP* genes were selected for the *cis*-regulatory element analysis using PlantCARE (http://bioinformatics.psb.ugent.be/webtools/plantcare/html/, accessed on 1 June 2023). The results were visualized with the BioSequence Viewer from TBtools [47]. MCScanX was used to analyze and identify the duplication events and collinearity relationships of the *PtMLP* gene family. The ratio of synonymous mutations to non-synonymous mutations (Ka/Ks) was calculated using Tbtools [47].

### 4.6. Expression Pattern Analysis of PsnMLP Gene Family

The expression patterns of the *PsnMLP* genes in different tissues (roots, stems, and leaves), under different treatments (salt, drought, and cold), were analyzed based on RNA-Seq.

Total RNA was extracted with an RNA extraction kit (Tiangen, Beijing, China) and subsequently reverse-transcribed into cDNA with the PrimeScript RT reagent kit (Takara, Beijing, China). Then, qPCR was conducted. Real-time PCR was performed on the CFX96 (Bio-Rad, Hercules, CA, USA) using TB Green Premix Ex Taq™ II (Takara, Beijing, China) with three biological replicates. The relative expression levels of the genes were calculated using the 2^−δδct^ method. Primers used in this research were listed in Table S4.

## 5. Conclusions

In this study, a total of 43 *PtMLP* genes distributed across 12 chromosomes were identified in the *Populus trichocarpa* genome. They could be classified into three subfamilies according to their gene structures and conserved motifs. Tandem duplications and segmental duplications played an important role in the formation of the *PtMLP* gene family, while these gene pairs underwent purification selection during the evolutionary process. The *PsnMLP* genes had different expression patterns, with most of them expressed in the roots at high levels and with two genes expressed only in the stems, while one was expressed only in the roots and one was expressed only in the leaves. In addition, the *PsnMLP* genes had different expression patterns under salt stress, cold stress, and drought stress, but members of the same subfamily had similar expression patterns. Most of the expression of the members of Class I and II was suppressed by abiotic stress, while most of the members of Class III were induced by stress. Class III included the majority of the genes exhibiting notable variations in expression before and after stress. *PsnMLP5* was chosen as a candidate gene for further research because it could react to all three abiotic stresses simultaneously. The findings indicated that *PsnMLP5* was responsive to ABA and may regulate poplar’s resistance to abiotic stresses by functioning within the ABA signaling pathway. The functional mechanisms of the poplar *MLP* genes in abiotic stress can be further investigated with the help of these findings, which are of great importance for poplar resistance breeding.

## Figures and Tables

**Table 1:** *PsnMLP* genes that exhibited significant variations in expression under abiotic stress.

GeneID	*PsnMLP* ID	Subfamily	Cold	Salt	Drought
Potri.001G405600	*PsnMLP1*	1		−4.4	
Potri.004G020100	*PsnMLP4*	2		−2.6	
Potri.004G021100	*PsnMLP5*	3	3.0	5.1	4.0
Potri.004G032900	*PsnMLP6*	3		−1.6	−1.1
Potri.004G033000	*PsnMLP7*	3		−1.3	−1.3
Potri.006G230600	*PsnMLP10*	1		−2.5	
Potri.008G131100	*PsnMLP12*	2	1.2	−1.0	
Potri.008G131200	*PsnMLP13*	2		−2.0	
Potri.008G131300	*PsnMLP14*	2		−1.1	
Potri.008G212100	*PsnMLP15*	3		10.6	
Potri.008G212400	*PsnMLP17*	3	1.3	2.5	
Potri.008G212500	*PsnMLP18*	3	1.4	2.5	
Potri.008G213100	*PsnMLP22*	3		3.1	1.2
Potri.010G000600	*PsnMLP28*	3			−1.2
Potri.010G111000	*PsnMLP30*	2		−2.2	−1.3
Potri.011G025900	*PsnMLP32*	3	2.9	3.4	
Potri.011G025966	*PsnMLP33*	3	2.9	3.7	
Potri.011G026032	*PsnMLP34*	3	1.9	3.3	
Potri.011G026100	*PsnMLP35*	3		4.0	
Potri.011G026200	*PsnMLP36*	3	3.0	3.5	1.3
Potri.013G131000	*PsnMLP38*	2		−2.29	
Potri.014G152800	*PsnMLP39*	3	2.2	2.9	1.5
Potri.016G046500	*PsnMLP40*	3	5.8	4.6	

The values represent the log_2_fold change under different treatments; blank indicates that there was no significant change in expression.

## Data Availability

Data is contained within the article and Supplementary Materials.

## Supplementary Materials

The following supporting information can be downloaded at: https://www.mdpi.com/article/10.3390/ijms25052748/s1.
