# Supplementary material for: Genome-Wide Characterization and Expression Analyses of Major Latex Protein Gene Family in Populus simonii × P. nigra
Source: Int J Mol Sci. 2024 Feb 27;25(5):2748. doi: 10.3390/ijms25052748 (PMC10931558; doi:10.3390/ijms25052748)
Supplement: Supplementary file 1 [file ijms-25-02748-s001.zip › ijms-2867753-supplementary.pdf]

Table S1. Characteristics of *MLP* family members in *Populus trichocarpa*

Sequence ID	ID	Number of Amino Acids	Molecular Weight	Theoretical PI	Instability Index	Aliphatic Index	Grand Average of Hydropathicity	Location
Potri.001G405600	PtMLP1	154	17311.64	4.76	25.93	84.03	-0.25	Cytoplasmic
Potri.003G088100	PtMLP2	156	17419.91	4.76	39.53	93.01	-0.26	Cytoplasmic
Potri.004G020000	PtMLP3	157	17771.2	8.70	41.58	83.12	-0.34	Nuclear
Potri.004G020100	PtMLP4	175	19994.8	7.59	38.78	81.83	-0.27	Nuclear
Potri.004G021100	PtMLP5	159	17593.13	5.15	36.12	85.97	-0.22	Cytoplasmic
Potri.004G032900	PtMLP6	162	17518.91	4.73	32.89	96.85	-0.003	Cytoplasmic
Potri.004G033000	PtMLP7	162	17557.9	4.65	28.77	95.00	-0.03	Cytoplasmic
Potri.004G051500	PtMLP8	159	18215.83	6.21	34.68	81.51	-0.50	Cytoplasmic
Potri.006G104100	PtMLP9	221	24059.16	8.73	44.12	84.57	-0.24	Extracellular
Potri.006G230600	PtMLP10	191	21248.02	5.43	34.04	89.69	-0.26	Cytoplasmic
Potri.008G073400	PtMLP11	214	23198.13	8.25	43.66	84.58	-0.22	Extracellular
Potri.008G131100	PtMLP12	150	17262.62	5.02	32.82	82.47	-0.52	Cytoplasmic
Potri.008G131200	PtMLP13	150	17043.57	5.96	35.73	88.33	-0.33	Cytoplasmic
Potri.008G131300	PtMLP14	150	16686.21	5.21	16.92	101.93	-0.10	Cytoplasmic
Potri.008G212100	PtMLP15	160	17762.34	4.88	30.51	93.19	-0.15	Cytoplasmic
Potri.008G212300	PtMLP16	157	17085.8	7.66	34.79	80.83	-0.19	Cytoplasmic
Potri.008G212400	PtMLP17	158	17635.32	5.20	34.86	87.59	-0.18	Cytoplasmic
Potri.008G212500	PtMLP18	158	17694.35	5.37	37.64	84.49	-0.26	Cytoplasmic
Potri.018G054400	PtMLP19	189	20950.77	5.62	39.79	88.10	-0.27	Cytoplasmic
Potri.008G212700	PtMLP20	156	16814.93	4.57	23.41	77.63	-0.20	Cytoplasmic
Potri.008G212923	PtMLP21	79	8471.61	4.54	28.15	77.85	-0.10	Chloroplast
Potri.008G213100	PtMLP22	186	21015.2	5.49	41.50	90.65	-0.13	Cytoplasmic
Potri.008G213223	PtMLP23	119	12792.45	4.54	28.36	77.14	-0.06	Extracellular/Chloroplast
Potri.008G213446	PtMLP24	156	16814.93	4.57	23.41	77.63	-0.20	Cytoplasmic
Potri.008G213669	PtMLP25	156	16814.93	4.57	23.41	77.63	-0.20	Cytoplasmic
Potri.010G000200	PtMLP26	160	17617.05	4.70	37.56	88.31	-0.17	Cytoplasmic
Potri.010G000400	PtMLP27	160	17571.02	4.70	34.57	90.12	-0.17	Cytoplasmic
Potri.010G000600	PtMLP28	158	17160.66	4.68	26.01	93.16	-0.08	Cytoplasmic
Potri.010G096000	PtMLP29	152	16989.51	5.19	22.78	89.67	-0.24	Cytoplasmic
Potri.010G111000	PtMLP30	158	17814.15	4.98	30.54	80.82	-0.30	Cytoplasmic
Potri.010G183900	PtMLP31	214	23276.36	7.67	54.69	87.76	-0.11	Extracellular
Potri.011G025900	PtMLP32	159	17602.17	5.31	34.95	87.80	-0.23	Cytoplasmic

Potri.011G025966	PtMLP33	159	17602.17	5.31	34.95	87.80	-0.23	Cytoplasmic
Potri.011G026032	PtMLP34	159	17656.2	5.19	36.06	88.99	-0.25	Cytoplasmic
Potri.011G026100	PtMLP35	159	17616.24	5.52	32.93	87.80	-0.23	Cytoplasmic
Potri.011G026200	PtMLP36	159	17612.23	5.52	34.51	90.25	-0.22	Cytoplasmic
Potri.011G127800	PtMLP37	190	21266.68	5.49	35.78	109.21	0.03	Mitochondrial
Potri.013G131000	PtMLP38	151	17156.48	4.89	42.97	74.90	-0.52	Cytoplasmic
Potri.014G152800	PtMLP39	159	17552.09	5.68	34.11	89.06	-0.21	Cytoplasmic
Potri.016G046500	PtMLP40	159	17768.39	5.16	41.16	87.17	-0.24	Cytoplasmic
Potri.016G125400	PtMLP41	221	24013.91	7.68	51.29	79.77	-0.32	Extracellular/Nuclear
Potri.017G051100	PtMLP42	153	16774.18	5.50	32.00	79.74	-0.07	Cytoplasmic/Chloroplast
Potri.017G051200	PtMLP43	120	13211.24	5.75	23.30	90.25	-0.06	Cytoplasmic

Table S2 Duplicated gene pairs of *PtMLP* gene family

**Tandem duplication**		**Segmental duplication**		**Duplication between** *Populus trichocarpa* and *Arabidopsis thaliana*	
Potri.004G020000	Potri.004G020100	Potri.004G021100	Potri.011G025900	At-rna-NM_129387.3	Potri-Potri.006G104100
Potri.004G032900	Potri.004G033000	Potri.006G104100	Potri.016G125400	At-rna-NM_129593.3	Potri-Potri.006G104100
Potri.008G131100	Potri.008G131200	Potri.006G230600	Potri.018G054400	At-rna-NM_128163.2	Potri-Potri.006G230600
Potri.008G131200	Potri.008G131300	Potri.008G073400	Potri.010G183900	At-rna-NM_129593.3	Potri-Potri.008G073400
Potri.008G212500	Potri.008G212400	Potri.008G131100	Potri.010G111000	At-rna-NM_120626.3	Potri-Potri.008G073400
Potri.008G212700	Potri.008G212923	Potri.008G213446	Potri.010G000600	At-rna-NM_001332591.1	Potri-Potri.008G131100
Potri.008G213223	Potri.008G213446	Potri.008G212500	Potri.010G000400	At-rna-NM_101362.3	Potri-Potri.008G131100
Potri.008G213446	Potri.008G213669	Potri.008G212100	Potri.010G000200	At-rna-NM_179544.2	Potri-Potri.008G131100
Potri.008G213669	Potri.008G212700			At-rna-NM_126213.3	Potri-Potri.008G131100
Potri.011G025900	Potri.011G025966			At-rna-NM_129593.3	Potri-Potri.010G183900
Potri.011G025966	Potri.011G026032			At-rna-NM_120626.3	Potri-Potri.010G183900
Potri.011G026100	Potri.011G026200			At-rna-NM_179544.2	Potri-Potri.010G111000
Potri.017G051100	Potri.017G05120			At-rna-NM_101362.3	Potri-Potri.010G111000
				At-rna-NM_001332591.1	Potri-Potri.010G111000
				At-rna-NM_126213.3	Potri-Potri.010G111000
				At-rna-NM_128163.2	Potri-Potri.018G054400

Table S3 Ka_Ks analysis result of MLP gene family between *Populus trichocarpa* and *Arabidopsis thaliana*

Seq_1	Seq_2	Ka	Ks	Ka_Ks	Effective Len	Average S-sites	Average N-sites	cN	cS	pN	pS	Note
At-rna-NM_129387.3	Potri.006G104100	0.26	NaN	NaN	615	153.50	461.50	102.33	123.67	0.22	0.81	High Sequence Divergence Value (pS>=0.75)
At-rna-NM_129593.3	Potri.006G104100	0.32	2.72	0.12	615	151.00	464.00	120.75	110.25	0.26	0.73	
At-rna-NM_128163.2	Potri.006G230600	0.21	NaN	NaN	561	132.42	428.58	78.67	118.33	0.18	0.89	High Sequence Divergence Value (pS>=0.75)
At-rna-NM_129593.3	Potri.008G073400	0.31	NaN	NaN	609	148.08	460.92	116.58	112.42	0.25	0.76	High Sequence Divergence Value (pS>=0.75)
At-rna-NM_120626.3	Potri.008G073400	0.31	NaN	NaN	603	147.83	455.17	116.00	117.00	0.25	0.79	High Sequence Divergence Value (pS>=0.75)
At-rna-NM_001332591.1	Potri.008G131100	0.59	2.09	0.28	438	92.58	345.42	140.83	65.17	0.41	0.70	
At-rna-NM_101362.3	Potri.008G131100	0.53	NaN	NaN	450	95.33	354.67	134.25	77.75	0.38	0.82	High Sequence Divergence Value (pS>=0.75)
At-rna-NM_179544.2	Potri.008G131100	0.56	1.59	0.36	450	96.75	353.25	140.17	63.83	0.40	0.66	
At-rna-NM_126213.3	Potri.008G131100	0.55	NaN	NaN	450	93.08	356.92	138.42	74.58	0.39	0.80	High Sequence Divergence Value (pS>=0.75)
At-rna-NM_129593.3	Potri.010G183900	0.30	NaN	NaN	612	148.92	463.08	112.83	116.17	0.24	0.78	High Sequence Divergence Value (pS>=0.75)
At-rna-NM_120626.3	Potri.010G183900	0.30	NaN	NaN	603	147.92	455.08	113.75	120.25	0.25	0.81	High Sequence Divergence Value (pS>=0.75)
At-rna-NM_179544.2	Potri.010G111000	0.36	NaN	NaN	471	99.58	371.42	107.33	75.67	0.289	0.76	High Sequence Divergence Value (pS>=0.75)
At-rna-NM_101362.3	Potri.010G111000	0.57	NaN	NaN	432	90.50	341.50	137.00	71.00	0.40	0.78	High Sequence Divergence Value (pS>=0.75)

At-rna-NM_001332591.1	Potri.010G111000	0.74	NaN	NaN	444	92.50	351.50	165.42	70.58	0.47	0.76	High Sequence Divergence Value (pS>=0.75)
At-rna-NM_126213.3	Potri.010G111000	0.74	NaN	NaN	453	91.50	361.50	170.67	70.33	0.47	0.77	High Sequence Divergence Value (pS>=0.75)
At-rna-NM_128163.2	Potri.018G054400	0.19	NaN	NaN	561	132.92	428.08	71.00	11.006	0.17	0.87	High Sequence Divergence Value (pS>=0.75)

Table S4 Primers used in this research

Primer name	Forward(5′-3′)	Reverse(5′-3′)
PsnMLP5-clone	TGAGTTTGCCATTGCTGTCG	GCTTCAATCATCTGCTTGGC
PsnMLP5-qRT	GCTCTAGAATGGGTGTCATCACTTCA	TCCCCCGGGCTAGGCATCTGGATT
PsnMLP36-qRT	GCCTGGAACCATCAGGAAGA	GCAAATGGATCCTCCCTCAG
PsnMLP39-qRT	CCTGGAGCCATCAGGAAGAT	GGAACCTCCCTCAGGAGTTG
